# Primary Malignant Liver Tumors: eight-year experience in a Pediatric Hospital in Brazil. A cross-sectional study

**DOI:** 10.1590/0100-6991e-20223273-en

**Published:** 2022-06-06

**Authors:** LORAINE ENTRINGER FALQUETO, PAULA RUBIO VILAR, HELDER GROENWOLD CAMPOS, CLAUDIO SCHULZ, ELISANGELA DE MATTOS E SILVA

**Affiliations:** 1 - Hospital Pequeno Príncipe, Cirurgia Pediátrica - Curitiba - PR - Brasil; 2 - Hospital Pequeno Príncipe, Radiologia - Curitiba - PR - Brasil

**Keywords:** Hepatectomy, Liver Neoplasms, Liver Transplantation, Pediatrics, Hepatoblastoma, Hepatectomia, Neoplasias Hepáticas, Transplante de Fígado, Pediatria, Hepatoblastoma

## Abstract

**Introduction:**

liver tumors are rare neoplasms in childhood (1-2%), and about 2/3 are malignant. Hepatoblastoma (HB) is the most frequent, followed by hepatocellular carcinoma (HCC). In both, the main treatment is surgical resection. Currently, chemotherapy and liver transplantation have improved outcomes.

**Objective:**

study of the epidemiological profile and evolution of liver cancer cases in a referral pediatric hospital.

**Methodology:**

a retrospective survey of medical records of patients aged up to 18 years with a diagnosis of primary malignant hepatic neoplasm between 2012 and 2020, carried out in the largest exclusively pediatric hospital in Brazil.

**Results:**

a total of 13 patients with malignant liver tumors (HB 12, HCC 1) were treated. Of the HB cases, 66,7% were male, with a mean age of 2 years and the main alteration in the palpable abdominal mass. Tumors involved an average of 3 liver segments, more in the right lobe (54%). Only one patient was treated with surgery without neoadjuvant therapy, another one underwent transplantation like the first treatment, and another 2 required liver transplantation as a rescue. The middle follow-up time of patients with HB was 39 months and only 1 case died due to febrile neutropenia. The 5-year overall and disease-free survival was 91.7% and 81.5%, respectively.

**Conclusion:**

Advanced staging at the time of diagnosis has always been a poor prognostic factor in patients with primary malignant liver tumors. However, the results and survival have improved with the advancement of chemotherapy, surgical technique, and liver transplantation.

## RESUMO

**Introdução:**

tumores hepáticos são neoplasias raras na infância (1-2%), sendo que cerca de 2/3 são malignos. O hepatoblastoma (HB) é o mais frequente, seguido do carcinoma hepatocelular (CHC). Em ambos, o principal tratamento é a ressecção cirúrgica completa. Atualmente, a quimioterapia e o transplante hepático têm melhorado os resultados.

**Objetivo:**

estudo do perfil epidemiológico e evolução dos casos de cânceres hepáticos em um hospital pediátrico de referência.

**Método:**

Levantamento retrospectivo de prontuários de pacientes até 18 anos com diagnóstico de neoplasia maligna primária hepática entre 2012 e 2020 realizado no maior hospital exclusivamente pediátrico do Brasil.

**Resultados:**

foram atendidos 13 pacientes com tumores malignos hepáticos (HB 12, CHC 1). Dos casos de HB, 66,7% eram do sexo masculino, com idade média de 2 anos e a principal alteração foi massa abdominal palpável. Os tumores envolviam em média 3 segmentos hepáticos, mais em lobo direito (54%). Um paciente foi tratado com cirurgia sem neoadjuvância, um foi submetido a transplante inicialmente e outros 2 necessitaram de transplante hepático como resgate. O tempo de seguimento dos pacientes com HB foi de 39 meses e apenas 1 caso foi a óbito por neutropenia febril. A sobrevida geral e livre de doença em 5 anos foi de 91,7% e 81,5% respectivamente.

**Conclusão:**

o estadiamento avançado no momento do diagnóstico sempre foi um fator de mau prognóstico em pacientes com tumores hepáticos malignos primários. Entretanto, os resultados e a sobrevida têm melhorado significativamente com o avanço da quimioterapia, da técnica cirúrgica e do transplante hepático.

## INTRODUCTION

Primary liver tumors represent less than 2% of neoplasms in pediatrics, at least two thirds of them being malignant^1^ ^,^ ^2^. The most frequent histological type is hepatoblastoma (HB) followed by hepatocellular carcinoma (HCC)^1^ ^,^ ^2^. In recent decades, an increase in the incidence of HB has been observed, accompanying the global epidemiological transition related to the higher prevalence of premature and low birth weight patients^2^ ^-^ ^4^.

Pediatric liver tumors are classified by the extent of disease before and after treatment with neoadjuvant chemotherapy, PRE Treatment EXTent of Disease (PRETEXT) and POST Treatment EXTent of Disease (POSTTEXT), respectively^5^. This evaluation is performed by the radiological image of the patient in the axial axis. This classification segments the liver into multiple sectors, ranging from I to IV. The more sectors involved, the more advanced the disease and the worse the prognosis. According to the Children’s Hepatic Tumors International Collaboration (CHIC), other characteristics also evaluated radiologically are added as aggravating factors regarding the progression and resectability of the disease, namely, the extension to other organs and great vessels, metastases, tumor rupture, multifocality, involvement of the caudate, and lymph node involvement^5^ ^,^ ^6^.

In general, the treatment of liver tumors is based on surgical resection with partial hepatectomy or, in some cases, liver transplantation. Chemotherapy is an excellent ally to treatment, improving outcomes, particularly in those patients with initially unresectable tumors^1^ ^,^ ^7^ ^,^ ^8^. New modalities of interventional radiology therapy have been discussed to help in specific cases with relapse or no response to neoadjuvant therapy.

The joint effort of surgeons, oncologists, hepatologists, and interventional radiologists contributes to a greater chance of cure. However, there are still divergent points in the literature regarding the treatment of pediatric liver cancer^8^. To better understand the evolution of the disease and treatment in the Brazilian reality, this work aims to study the epidemiological profile and evolution of liver cancer cases in an exclusively pediatric referral hospital in southern Brazil.

## METHODS

After approval by the Ethics in Research Committee under opinion 4.268.222, we carried out an observational, longitudinal, descriptive, and retrospective study from the survey of the electronic medical records of patients aged up to 18 years treated between June 2012 and December 2020, diagnosed with malignant liver disease, at Pequeno Príncipe Hospital.

Epidemiological data were tabulated in a Microsoft Excel database. The imaging exams with images available in the system were reassessed by the radiology staff for review and PRETEXT and POSTTEXT reclassification.

We performed a general descriptive analysis, expressed as absolute frequency and relative percentage for categorical variables, as well as mean and standard deviation for continuous ones. We calculated the rate of surgical complications for patients with HB. Furthermore, the response to chemotherapy based on the radiological volume of the lesion was expressed as an average percentage of tumor reduction with neoadjuvant treatment in cases in which it was applied. We also calculated overall and disease-free survival of patients with HB, expressed as probability of survival over time, with the Kaplan-Meier method. The survival curves were calculated using the R (Version 3.6.3) and Rstudio softwares (Version 2021.09.0 Build 351).

## RESULTS

Between 2012 and 2020, 13 patients with malignant liver tumors were treated at the Pequeno Príncipe Hospital in Curitiba, 12 with HB and one with HCC. Among the patients with HB, the majority (66.7%) were male, with a mean age at diagnosis of two years and four months. The most frequent clinical presentation was an abdominal mass palpable by family members (Table 1).

**Table 1:** Epidemiologic profile.

Tumor	Hepatoblastoma (n=12)	Hepatocarcinoma (n=1)
Mean Age	2y 4m (SD 12.65 m)	6y 6m
Sex	M = 66.7% (8)	M
	F = 33.3% (4)	
Prematurity	2 (28 and 34 weeks)	0
Associated diseases	Coarctation of aorta (1)	Type-1 tyrosinemia
	Aortic valve stenosis (1)	Cirrhosis
	Esophageal atresia (1)	
Tumor	Hepatoblastoma (n=12)	Hepatocarcinoma (n=1)
	Congenital clubfoot (1)	
	Single umbilical artery (1)	
Symptoms	Palpable mass - 7 (58%)	Abdominal distension Inadequate weight gain
	Abdominal pain - 4 (33%)	
	Abdominal distention - 3 (25%)	
	Anemia; Fever; Weight loss (2 occurrences for each symptom - 16%)	
	Early satiety; Finding at radiological exam; Inappetence; Digestive bleeding; Jaundice; Diarrhea (1 occurrence for each symptom - 8%)	
Death	1	1

a: years; m: months/ SD: standard deviation; F: female; M: male.

Only two patients with HB had a history of prematurity. Of the 13 patients, some congenital malformations were observed among the patients with HB, with a predominance of cardiovascular alterations (Table 1).

Images from 11 patients were available for review by the radiologist. The mean tumor volume at diagnosis was 439cm³ (SD 82.7cm³), involving an average of three liver segments, with a predominance of the right lobe (54%); in three cases the involvement was bilateral. Of the patients undergoing chemotherapy, it was possible to compare images before and after neoadjuvant therapy in nine cases, with a mean reduction of 63% (SD 15.5%) in lesion size. The PRETEXT classification is summarized in Table 2.

**Table 2:** Tumor classification.

Tumor	Hepatoblastoma (12)	Hepatocarcinoma (1)
Average AFP	83,603 (SD 30,988)	125.120
PRETEXT	I - 3 (25%)	I (100%)
	II - 4 (33%)	
	III - 5 (41%)	
NI	1	0
Vena cava	3 (25%)	
Portal Vein	2 (16%)	
Caudate	1 (8%)	
Extrahepatic extension	5 (41%)	
Rupture	1 (8%)	
Metastasis	2 (16%)	
Histologic subtype	Embryonic comp. = 5	
(n=10 - with detailed information)	Fetal comp. = 3	
	Mesenchymal comp. = 5	
	Teratoid comp. = 2	
	Rhabdomyoblastic comp. = 1	

AFP: alpha-fetoprotein; NI: not informed; NA: not applicable; comp.: component.

Alpha-fetoprotein (AFP) mean value at diagnosis was 83,603 among patients diagnosed with HB, ranging from 1,398 to over 300,000. The most frequent histological subtypes were epithelial with an embryonic component (50%) and mesenchymal (50%); 90% of the tumors had mixed histology.

The access route for biopsy was through laparotomy in most cases, 43% (n=5), with a higher prevalence of less invasive techniques in recent years (Table 3).

**Table 3:** Surgical approaches.

Tumor	Hepatoblastoma (12)	Hepatocarcinoma (1)
Biopsy	83% (10)	Yes
Open (wedge)	42% (5)	100%
Percutaneous	25% (3)	
Laparoscopic	16% (2)	
Chemotherapy		No
Neoadjuvant	83% (10)	
Adjuvant	75% (9)	
Surgery		non-operated
Left hepatectomy	2	
Right hepatectomy	4	
Right Bi-Seg	2	
ALPPS	3	
Transplant	1	
Pulmonary Seg	3 (4)*	
Reoperation (non-oncological cause)	1 (8%)	
Rescue surgery		
Transplant	2	

ALPPS: Associating liver partition and Portal vein Link for Staged hepatectomy; Seg.: Segmentectomy; (*) three patients underwent lung segmentectomy for metastasis; of those, one underwent bilateral surgery, totaling four procedures.

In the years 2013, 2016, and 2017, three cases underwent Associating Liver Partition and Portal vein Ligation for Staged Hepatectomy (ALPPS) due to less than 25% disease-free residual liver parenchyma, caudate lobe involvement, and portal invasion. These patients progressed well, but two of them also required thoracotomy for excision of lung metastasis (Table 3).

Of the three patients who required thoracotomy for lung metastasis, two were after ALPPS surgery. The third patient had bilateral lung metastasis after left hepatectomy and underwent left thoracotomy after six months and right thoracotomy three weeks after the initial surgery.

After surgery, patients remained on mechanical ventilation for an average of two days and in the intensive care unit for 5.6 days.

The early surgical complication rate was 41.7% (Table 4). Only one patient was reoperated for hypovolemic shock, though with no active bleeding found in the reapproach procedure. The rate of late surgical complications was 8.3% (n=1, incisional hernia).

**Table 4:** Clinical and surgical complications.

Surgical complications
Hemorrhagic shock (3)
Subphrenic abscess (1)
Biliary fistula (1)
Incisional hernia (1)
Acute clinical complications
Convulsive crisis (1)
Hypotension (1)
Hypoglycemia (1)
Catheter-associated infection (1)
Urinary infection (2)
Acute rejection (1)
EBV infection (1)
Pleural effusion due to hypoalbuminemia (1)
Long-term clinical complications
Retinal detachment
Thrombocytopenia
CMV infection

EBV: Epistein-Barr virus; CMV: Cytomegalovirus.

Only one case underwent surgery as an upfront treatment (PRETEXT I). The other patients with HB received neoadjuvant chemotherapy followed by surgery. Only two did not receive adjuvant chemotherapy.

During chemotherapy after hepatectomy, tumor recurred in two cases, which were referred for liver transplantation as salvage therapy. One of them was transplanted at another service and was lost to follow-up.

**Figure 1:**
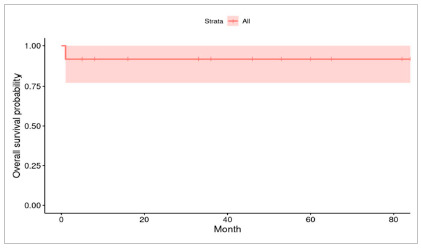
Five year Overall Survival Curve for patients with HB.

**Figure 2:**
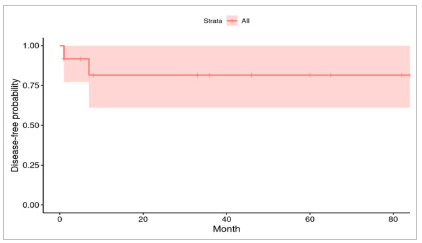
Five year disease-free survival curve for patients with HB.

The follow-up time of patients with HB was on average 39 months, ranging from four months to six years and ten months, until December 2020, with a five year overall survival of 91.7% and a five year disease-free survival of 81.5%. Figures 1 and 2 depict the survival curves. The HCC case was not included in this analysis.

Only one death was recorded among patients with hepatoblastoma, not directly related to surgery. The patient evolved with neutropenia during adjuvant chemotherapy, severe pulmonary hemorrhage, and death.

A single patient with hepatocarcinoma was identified in this study, diagnosed during follow-up of hepatic cirrhosis related to Type 1 Tyrosinemia. However, due to failure to adhere to treatment and social conditions, hepatocarcinoma had a late diagnosis, with advanced liver disease and esophageal varices. It was confirmed by ultrasound-guided percutaneous biopsy and this patient died due to digestive bleeding, considered a clinical complication related to the disease evolution.

## DISCUSSION

Liver tumors can be classified as epithelial - hepatocellular and biliary, mesenchymal, metastatic, and rare subtypes - germ cell and rhabdoid tumors^1^. The most frequent primary malignant tumors in pediatrics are hepatocellular neoplasms, HB and HCC^1^. Less than 10% of childhood hepatic malignancies correspond to metastases, mesenchymal tumors (sarcoma, angiosarcoma, and epithelioid hemangioendothelioma) and biliary tumors (cholangiocarcinoma)^9^.

HB is the most prevalent hepatic malignancy. In the eight years of follow-up, 92% of the cases were HB. Despite a slight predominance of males in this analysis, the frequency is approximately equal between sexes, mostly in younger children, between six months and four years of age^3^.

Generally, cases are sporadic, but some forms are associated with genetic syndromes^3^. We observed no associated genetic diseases, although we noticed the presence of cardiopathies and other perinatal malformations. Publications have shown an increase in the incidence of HB, following the global epidemiological transition, with a higher prevalence of premature and low birth weight patients. Other known risk factors are exposure to drugs, plastics, radiation, metals, and tobacco^2^.

More than half of the patients present with an abdominal mass on palpation, either perceived by the pediatrician or by family members, and with nonspecific symptoms linked to abdominal distension. The perception of family members should be valued, especially if accompanied by other constitutional symptoms. Less frequent compressive symptoms may be related to a worse prognosis location or very large tumors, causing jaundice, portal hypertension, and compression of hollow viscera^1^.

Unlike HB, HCC predominantly affects male adolescents and has a lower survival rate^7^ ^,^ ^8^ ^,^ ^10^. The incidence varies depending on geographic factors and the prevalence of hepatitis B in the community^7^ ^,^ ^10^ ^,^ ^11^. Lesions may be sporadic or preceded by underlying liver disease, such as liver cirrhosis or metabolic, infectious, or vascular disorders^7^. Among these, the main risk factors are tyrosinemia and perinatal Hepatitis B^10^ ^,^ ^11^. The only case of HCC in this series received an early diagnosis of tyrosinemia, but with low adherence to treatment even in the face of social service interventions. This was the main factor responsible for the poor evolution of the patient during the entire follow-up at the institution.

Regardless of the histological subtype, from the suspicion of a liver tumor the investigation begins with an ultrasound examination of the abdomen^1^ ^,^ ^9^ ^,^ ^11^ ^,^ ^12^. Axial images, preferably magnetic resonance imaging (MRI), provide more details on location, size, resectability, and probable tumor etiology. MRI with hepatocyte-specific contrast is the best radiological modality for diagnosis in association with elevated AFP^1^ ^,^ ^12^. Although AFP is a marker of hepatocytic neoplasms, its increase is not a necessary condition for diagnosis^2^ ^,^ ^9^. Furthermore, normal AFP may mean a worse prognosis neoplastic behavior^2^ ^,^ ^5^. In addition to the abdominal examination, the search for metastatic lesions is essential, chest tomography being part of staging^8^ ^,^ ^9^ ^,^ ^12^.

Tumor biopsy is the gold standard and necessary in pediatric patients for therapeutic definition, type determination, histological grade, and genomic study^11^ ^-^ ^13^. Currently, ultrasound-guided needle biopsy performed by an interventional radiologist or trained pediatric surgeon is safe and effective in diagnosis. About five to 10 fragments of 1x0.3cm are needed for adequate sampling^2^. In cases where image-guided percutaneous biopsy is not possible, laparoscopy or laparotomy are alternatives that guarantee biopsy under direct vision, with vascular control using energy. Care must be taken in choosing the incision site so that it is included in the subsequent curative surgery^2^.

After the histological diagnosis, based on the number of affected liver segments and the involvement of other structures, the PRETEXT staging is performed, that is, the risk classification before treatment. According to Children’s Oncology Group (COG), tumors located in up to one segment (PRETEXT 1) and with radiological margin of at least 1cm from the great veins can be resected without neoadjuvant therapy^2^. As for the European group, Société Internationale d’Oncologie Pediatric (SIOP), all patients should be submitted to neoadjuvant therapy and reassessed after two cycles with new tests (POSTTEXT)^2^. This classification extends to both hepatoblastoma and hepatocarcinoma. Due to the limitations of the retrospective study, we could not access images from all patients. Reclassification after radiologist review only included nine out of 12 patients with HB. In these, we observed a mean reduction in lesion size of 63%, which increased the possibility of total resectability of the lesion, essential for healing, as well as reinforcing the adequate responsiveness of these tumors to chemotherapy. The reduction of AFP and tumor size after chemotherapy by 60% and 50%, respectively, has a direct correlation with prognosis^14^.

As previously mentioned, partial hepatectomy is the main surgical treatment modality. In planning, it is important to assess whether the healthy hepatic residual area is sufficient. At least 25% of the liver must be preserved so that the patient does not suffer from liver failure until it is regenerated. ALPPS surgery was a good alternative for patients with unresectable lesions with insufficient residual area and without quick access to liver transplantation. In this technique, the staged section of the liver allows the healthy residual area to undergo enough hypertrophy in the interval between surgeries. Laparoscopic and robotic surgical access is possible in liver resections. However, the technical skills required for these procedures display a steep learning curve. Furthermore, robotic surgery is not yet widely available in pediatric surgery.

Early diagnosis and good response to chemotherapy have guaranteed good results. However, some patients present with unresectable lesions even after neoadjuvant therapy. For these, primary liver transplantation proved to be an alternative with better survival, compared with wide resections^2^. A retrospective analysis comparing the indication of liver transplantation as primary and as salvage treatments found better results when transplantation is chosen early instead of radical liver resections^15^ ^,^ ^16^.

The lung is the main site of metastases and up to 17% of patients present this finding at diagnosis^6^. Three patients had lung metastasis and required thoracotomy for surgical resection after liver surgery and chemotherapy.

Metastectomy and new minimally invasive procedures performed by the interventional radiologist are important in salvage treatment^2^. Radiofrequency Ablation (RA) and Transarterial Chemoembolization (TACE) are examples of treatments that help control symptoms and reduce lesion size^2^ ^,^ ^9^ ^,^ ^17^ ^,^ ^18^. These techniques are already used as bridge therapy for adult patients and, though still not widespread for pediatric ones, should be considered. A retrospective study showed no differences in safety and in the response rate to RA in relapsed HB compared with surgery, as well as an alternative to tumors that did not respond to neoadjuvant therapy^18^ ^-^ ^22^. Specialized treatment centers with radiology, oncological surgery, and liver transplantation teams must be at the forefront of treatment, together and early.

Patients undergoing chemotherapy are immunodeficient and thrombocytopenic, which increases the risk of early surgical and clinical complications, such as bleeding, abscess, dehiscence, fistulas, and hernias, as observed. They should be prepared with adequate nutritional and clinical monitoring throughout the perioperative period.

Outcomes have greatly improved with clinical and surgical advances. According to Aronson et al., stage I patients have an estimated five year survival rate of 90%. However, patients with metastatic disease still have a variable and poor outcome, with survival between 45-80%^3^. Moreno et al., in a 15 year survey of the pediatric population in Argentina, reported a five year survival rate of 74.7% and 35.3% for HB patients with localized and metastatic disease, respectively^23^. The survival observed in this study was compatible with the literature, being 91.7% overall and 81.5%, disease-free.

In a retrospective study analyzing factors related to survival in HB, Karalexi et al. concluded that age over eight years and AFP below 1,000mg/ml are factors associated with reduced five year survival, from 80% to below 50%^5^ ^,^ ^24^. In our sample, only one patient was older than eight years (13), but the exams were not available for review. In any case, the patient evolved well with a three year follow-up without recurrence or complications so far. No patient with available data had an AFP below 1,000mg/ml.

In general, the recurrence rate of HB is 12%^23^. Uchida et al. presented a five year overall survival of 80.9% of 100 patients with HB submitted to transplantation, which was the primary treatment in 69.4% of individuals^15^. However, patients with locally advanced disease (PRETEXT IV) and metastasis at diagnosis maintain low survival rates, despite neoadjuvant chemotherapy and lesion resectability, with an overall five year survival rate of 51-64%^24^.

The treatment of HCC differs from that of HB, as the former presents a limited response to chemotherapy compared with the latter^2^. These variations are important in decision-making and surgical procedures. The main objective of staging is to assess the resectability of the lesion, including assessment of remaining liver tissue, tumor location, and number and size of lesion(s).

Partial hepatectomy is indicated in hepatocellular carcinoma for children with a localized liver mass, while individuals with multiple lesions with associated liver disease and who meet the Milan criteria are more likely to be referred to liver transplantation^1^ ^,^ ^7^ ^,^ ^8^. In the experience with the patient who was already cirrhotic and had HCC, the possibility of cure with liver transplantation was limited by social issues. Clinically, there were no conditions for other non-curative possibilities, such as hepatectomy and embolization, due to the advanced degree of cirrhosis caused by tyrosinemia. Unfortunately, only 20% of pediatric patients with HCC are candidates for surgical treatment at diagnosis^2^.

Advances in interventional radiology and the reestablishment of liver transplantation in the hospital from January 2020 onwards were considered a milestone in the service, given that they allowed transplantation as an initial treatment for one patient and rescue therapy for another, quickly and in the ideal period after indication. These resources are essential for the treatment of pediatric liver cancer and highlight the importance of referral centers for early treatment and follow-up from diagnosis^2^ ^,^ ^9^ ^,^ ^15^ ^,^ ^19^ ^,^ ^20^. Unfortunately, the follow-up time after this milestone is insufficient to allow comparisons between periods.

Liver neoplasms are rare, making it difficult to compare the results with other world series. The effort of the Brazilian scientific community to develop multicenter studies and the creation of a national database is essential for the strengthening of services, data analysis, and, mainly, for the improvement of patient care and survival.

A major international clinical trial has been ongoing since 2017 and is expected to be completed in December 2022, the Pediatric Hepatic International Tumor Trial (PHITT)^25^ ^,^ ^26^. This study mainly evaluates the risk stratification proposed by CHIC based on PRETEXT and AFP, as well as the survival of the groups according to the proposed lines of treatment. Its results will bring many other answers and a uniform direction regarding the treatment of both HB and HCC in pediatrics.

## CONCLUSION

Due to the low frequency of malignant liver tumors, the dissemination of the experience of each center is important for the construction of uniform treatment protocols, with good outcomes. Less invasive diagnostic procedures have been allies in reducing patient morbidity and mortality. Borderline patients for curative treatment should be referred as soon as possible to specialized centers with availability of liver transplantation, increasing the chance of cure and better results.
